# Cities as complex systems—Collection overview

**DOI:** 10.1371/journal.pone.0262964

**Published:** 2022-02-25

**Authors:** Diego Rybski, Marta C. González

**Affiliations:** 1 Potsdam Institute for Climate Impact Research – PIK, Member of Leibniz Association, Potsdam, Germany; 2 Department of Environmental Science Policy and Management, University of California Berkeley, Berkeley, CA, United States of America; 3 Complexity Science Hub Vienna, Vienna, Austria; 4 Department of City and Regional Planning, University of California Berkeley, Berkeley, CA, United States of America; PLOS, UNITED KINGDOM

## Abstract

This collection provides a contemporary excerpt of “Cities as complex systems”. The contributions have been submitted between April and October 2020. We briefly discuss example papers addressing the themes “urban scaling”, “urban mobility”, “flows in cities”, “spatial analysis”, “information technology and cities”, and “cities in time”. After motivating the intersection of cities and complexity, we provide an introduction and additional thoughts on urban scaling.

## Introduction

Cities represent the stage for a wide range of human activities including services, government, education, commerce, markets, finance, etc. Today they are the dominant form of living even while the process of urbanization is ongoing. However, the history of cities was not always a success story and often they are associated with traffic jams [1], pollution [2], health impacts [3], diseases [4], crime [5], inequality [6], and informal settlements [7]. Nevertheless, cities are attractive to people despite all these downsides.

Many of these negative characteristics can be attributed to difficulties in managing cities which in turn is due to their high level of complexity. Cities consist of interacting entities, such as people and infrastructure, and exhibit emergent properties, which appear additionally to the sum of the isolated properties [8]. Understanding individuals, e.g. in terms of psychology or economics, is usually not sufficient to understand cities as a whole [9]. This makes cities a prime example of complex systems. Accordingly, complexity theories and methods are particularly relevant to their study. “Cities are seen around the world and through history, suggesting ‘universal’ reasons for their existence” [10]. Such reasons suggest that as first step in their study one can focus on what they have in common before paying attention to what distinguishes the individual cities. Common mechanisms represent a key-premise of urban complexity research.

There are various arguments for treating cities as complex systems [8]. First, they can be seen as a superposition of social, physical, and virtual networks [11, e.g.], with features and data having lattice or grid character. Second, discrete dynamics and cellular automata are successfully used to describe urban dynamics [12, e.g.]. Third, the dynamics can also be continuous in the form of dynamical systems [13, 14, e.g.]. Fourth, agent-based modeling permits the study of individuals and their interactions in cities [15, e.g.]. Fifth, an important issue is the question of scale—or rather the absence of a characteristic scale.

Scaling is closely related to the concept of *scalability* as known in computer science. Bondi [16] describes “… a system as having space-time scalability if it continues to function gracefully as the number of objects it encompasses increases by orders of magnitude”. Cities fulfill this definition as the population size ranges from thousands to millions. Accordingly, we can draw the analogy of cities as a scalable system. However, this scalability requires some degree of adaptation, e.g. metropolises usually need an efficient public transport system like subways. Next, we would like to illustrate consequences of such adaptation.

A typical everyday-life example could be the following. “Imagine you want to buy 2.5 kg of potatoes. One kg of potatoes costs 1.50 Euro. Then half a kg of potatoes costs 0.75 Euro and 2.5 kg cost five times as much (3.75 Euro)”. Empirical analysis has shown that for many urban measures such a linear proportionality does not hold. E.g. doubling city size leads to approximately 2^1.15^ ≈ 2.2 times the total urban GDP, i.e. 20% more (see below). This means one city generates more GDP than two cities of half the size together. Consequently, the above exercise from the daily life *does not work* for city systems.

One can distinguish two types of scaling among cities. First, the number of cities scales inversely with the population size. It is commonly referred to as “Zipf’s law for cities” [17–19] and captures that there are many more small cities than large ones across orders of magnitude. Meaning, the probability of finding a city of size *S* is inversely proportional to its size. Settlements and cities cover a large range of spatial and size scales. Second, many urban indicators scale non-linearly with population size. This relation is often termed “urban scaling” [13, 20] and captures e.g. the non-proportionality of scalability for cities, as illustrated by the example above. For GDP it implies an “extra-wealth” of large cities which is one of the reasons why people are attracted despite their negative properties. This phenomenon is also known as “increasing returns to scale.” Imagine a farmer who can produce 5 tons of potatoes. The farm of her or his neighbor is 10× larger and accordingly can afford more efficient technology and machinery—so that the yield is e.g. 70 tons (instead of 50 tons). In cities, high densities of population and infrastructure lead to “agglomeration effects”, which not only explain increasing returns but according to urban economics also the existence of cities in the first place [21–23].

This collection provides a contemporary excerpt of “Cities as complex systems”. The contributions have been submitted between April and October 2020—we received over a 100 submissions and PLoS One published over 60. They can be classified into a set of themes. In the following we want to briefly discuss examples. Our choice is of course subjective and the collection includes other interesting papers.

## Urban scaling

Ortman, Lobo, and Smith [24] take up the idea of cities as complex systems and discuss what ancient and present cities have in common. They reason how cities can be defined and delineated. From agglomeration effects the authors make the connection to urban scaling between population size and urban area and motivate a theoretical exponent 5/6 implying that a city of double the size has a 12% higher density than two cities of half the size. The theoretical exponent is compared to the empirical analysis of ancient and present day urban systems where in many cases an exponent smaller than 1 is confirmed. The authors conclude that there are always empirical challenges and that predictive theory is necessary for the larger goals of urban science.

## Urban mobility

A planned community is one whose layout exhibits a clearly recognizable plan or system. Yinger [25] compares two examples, namely Black Rock City and the Manhattan borough. Black Rock City is designed and built every year to host the Burning Man Festival in the Nevada desert attended by tens of thousands of people. Specifically, the author compares—using the tools of urban economics—the circular layout of Black Rock City and the well-known rectangular grid of Manhattan. He finds that cities with circular layout have higher population densities given the same length or radial commuting lines. This result can swap in case of a large number of radial lines or expensive access to them. Radial lines diagonal to the rectangular grid lead to cities with lower densities.

## Flows in cities

Peréz-Mendéz et al. [26] study reversible lanes which are intended to alleviate traffic congestion in cities. The direction of such lanes can be reversed in the more congested direction for a limited period. The reasoning is that a large share of vehicle infrastructure is underused because of the asymmetries on travel demand. The authors employ cellular automata to investigate adaptive reversible lanes, which respond to real-time traffic demand. Analyzing real-world traffic flows, the authors find strong fluctuations even during rush hours. Their analysis shows that relative to conventional reversible lanes, adaptive ones can be up to 40% more efficient. However, Peréz-Mendéz et al. also mention that the precise advantage depends on a set of factors, including the variability of traffic, the implementation of reversible lanes, and city specifics. Finally, challenges are discussed, in particular of safely operating adaptive lanes.

## Spatial analysis

Silver, Byrne, and Adler [27] extend the Schelling model of segregation. The authors argue that many human interactions take place in different venues such as: offices, schools, stores, bars, parks, religious centers, etc. After discussing physical features, catchment area, mandatoriness, and openness of a venue, the authors introduce it in the classical Schelling model via travel distance, openness, and mandatoriness and use a Moore neighborhood distance. From their agent-based modeling they report that venues make segregation less likely when the agents are relatively tolerant and more likely if they are intolerant. Multiple venues can lead to structures beyond their catchment areas. The authors conclude that the types and locations of venues in cities could play an important role for the stability or segregation in cities.

## Information technologies and cities

The idea of Mulisheva et al. [28] is to use Twitter data, which is freely available, as an information source for urban planning and development. Specifically, the authors consider traffic accidents in Nairobi, Kenya, during the period 2012–2020. Therefore, they extract almost one million tweets and apply machine learning in combination with geoparsing in order to obtain the occurrence and location of the events. The identified crashes have been validated on the ground by a motorcycle delivery service that was sent in real-time—resulting in 92% accuracy. The authors produce the first map of accidents for Nairobi, which is particularly valuable given the high mortality rates of children and adolescents and given the resource-poor environment. The authors conclude that mining Twitter data can support the design of enforcement policies and infrastructure development.

## Cities in time

Xie et al. [29] analyze the land-cover evolution of Chinese cities. Specifically, they combine nightlight and Landsat data and, as an example, study the three northeastern provincial capitals Shenyang, Changchun, and Harbin. The proposed multi-source approach improves the accuracy compared to single-source data methods. The authors then use class area, percentage of land, fragmentation index, and aggregation index to characterize the land-cover evolution. As expected the absolute impervious surface of all three cities increased across the years 2000, 2004, 2008, and 2012. However, the vegetation also increased and the relative share remained approximately constant except for Harbin, where the share of vegetation slightly increased. Despite this growth, the number of patches relative to the total area decreased in all cases. The authors conclude that the land-use efficiency in the main urban areas is improving, and Shenyang and Harbin are doing better in ecological terms.

Beyond this collection, Arcaute & Ramasco [30] provide a synthetic review of the field, employing similar categories as those above. The authors argue that “many of the spatial correlations of the different processes taking place in cities, are tightly related to the spatial distribution of functions and transport, which are both closely linked to the morphology of cities”—and these interdependencies are poorly understood. They conclude that the time has come to couple the various disciplines treating cities that emerged over the last century.

## Closing

Cities are and have been fundamental to our civilizations for centuries. If we want to sustain urban living in view of present and future challenges, then we need to understand them. This means they need to be treated as what they are: complex systems. With a considerable number of papers, here we could only introduce a minor subset, this collection covers a range of topics around cities as complex systems. Thereby, it not only advances the field but also contributes to the establishment of the urban science discipline [31–33]. In this spirit, the collection encourages the formation of a scientific community that collaborates in publications and meetings in order to drive the scientific progress and understanding of urban systems.
